# Human Fungal Infections in Kuwait—Burden and Diagnostic Gaps

**DOI:** 10.3390/jof6040306

**Published:** 2020-11-21

**Authors:** Wadha Alfouzan, Faten Al-Wathiqi, Haya Altawalah, Mohammad Asadzadeh, Ziauddin Khan, David W. Denning

**Affiliations:** 1 Microbiology Unit, Department of Laboratories, Farwaniya Hospital, P. O. Box 13373, Farwaniya 81004, Kuwait; Alfouzan.w@ku.edu.kw; 2 Department of Microbiology, Faculty of Medicine, Kuwait University, P. O. Box 24923, Safat 13110, Kuwait; Haya.altawalah@ku.edu.kw (H.A.); Mohammad.assadzadeh@ku.edu.kw (M.A.); zkhan@hsc.edu.kw (Z.K.); 3 Department of Medical Laboratory Sciences, Faculty of Allied Health Sciences, Kuwait University, P. O. Box. 31470, Sulaibikhat 90805, Kuwait; 4 Virology Unit, Department of Laboratories, Yacoub Behbehani Center, Sabah Medical Area, P.O. Box 4078, Shuwaikh 13001, Kuwait; 5 Manchester Fungal Infection Group, The University of Manchester and the Manchester Academic Health Science Centre, Manchester M13 9PL, UK; ddenning@manchester.ac.uk

**Keywords:** mycoses, epidemiology, aspergillosis, invasive fungal infections, dermatomycoses, Kuwait

## Abstract

Fungal infections are an increasingly important public health issue, yet accurate statistics on fungal burden worldwide and in Kuwait are scarce. Here we estimate the incidence and prevalence of fungal infections in Kuwait. Population statistics from 2018 collected by the Public Authority for Civil Information were used, as well as data from the Ministry of Health. A literature search for Kuwait data on mycotic diseases and population at risk (chronic obstructive pulmonary disease, HIV infection/AIDS, cancer, and transplant patients) was conducted. The population in 2018 was estimated at 4,226,920 million people: 1,303,246 million Kuwaitis and 2,923,674 million expatriates. We determined the annual burden of serious fungal infections number (per 100,000) from high to low based on earlier reported fungal rates for populations at risk: recurrent *Candida* vaginitis 54,842 (2595); severe asthma with fungal sensitisation 10,411 (246); allergic bronchopulmonary aspergillosis, 7887 (187); chronic pulmonary aspergillosis 995 (21.3); invasive aspergillosis 704 (16.7); fungal keratitis 654 (15.5); candidaemia 288 (6.8); *Candida* peritonitis 63 (3.5) and oesophageal candidiasis in HIV 33 (0.8). Besides identifying rising new risk groups and expanding reports on antifungal resistance, surveillance programs and further epidemiological studies are needed to achieve more precise assessments of fungal disease epidemiology and correlated morbidity and mortality.

## 1. Introduction

Fungal infections have long been considered of little importance to public health because most are not transmissible (*Pneumocystis* and dermatophytes being exceptions), and antifungal resistance has been until recently a relatively minor problem [1]. The priority has recently changed with the acceptance of several skin infections as Neglected Tropical Diseases, increased emphasis on advanced human immunodeficiency virus (HIV) infection and preventing deaths, and increasing antifungal resistance [1]. The emergence of *Candida auris* as a new multidrug-resistant nosocomial pathogen has highlighted the problem. Other major concerns concerning antifungal resistance are azole resistance in *Aspergillus fumigatus* and *Candida parapsilosis*, multidrug resistance in *Candida glabrata*, and terbinafine resistance in dermatophytes [2,3,4,5].

Fungal infection carries considerable mortality and morbidity in addition to a major financial impact on health care systems [6]. Accurate information and statistics on fungal burden worldwide and the Middle East are scarce. Although there have been major improvements in diagnostic technologies for both invasive and chronic fungal infections in the last decade, these assays are not yet widespread. Without them, the incidence and prevalence of fungal disease have been grossly underestimated. Multiple individual country estimates have been published using similar methodologies, as well as some international or global estimates—all indicative of well over 1.5 million deaths from fungal disease globally [1,6]. This high number is explained by the rise of at-risk populations including asthma, tuberculosis, acquired immunodeficiency syndrome (AIDS), chronic obstructive pulmonary disease (COPD), cancer, and immunosuppressed patients on chemotherapy [6,7,8,9]. Many factors such as socioeconomic conditions, hospital infection practices, unrestrained use of corticosteroids and antibiotics, geographic region, and cultural habits contribute to a marked variability of the incidence and prevalence of fungal disease worldwide [8,10,11,12,13,14,15].

Over the past 5 years, the Leading International Fungal Education (LIFE) portal has assisted in the estimation of the burden of serious fungal infections in more than 60 countries covering over 5.7 billion people worldwide [16]. Interestingly, pronounced differences are observed between countries and even within regions of the same country. However, many countries have no such data published, including Kuwait. 

Global and national data comparisons were analysed by Bongomin et al. in a review where they aggregated estimates of certain fungal infections’ annual incidence including the following: cryptococcal meningitis complicating HIV/AIDs was around 223,100; invasive candidiasis (IC) was 700,000, of which 100,000 were related to intra-abdominal infections; invasive aspergillosis was >300,000 from 10 million at risk annually; and *Pneumocystis jirovecii* pneumonia was 500,000. Prevalence estimates included chronic pulmonary aspergillosis (CPA) at 3,000,000, fungal asthma at >10,000,000, and over 1 billion of superficial fungal infections [1]. These large numbers of affected individuals together have a major financial and healthcare impact as well as leading to high mortality rates associated with invasive fungal infections, reaching up to 50% despite the availability of antifungal drugs [17,18]. 

Kuwait is a Middle Eastern country situated on the northern edge of Eastern Arabia at the tip of the Arabian Gulf. It shares borders with Iraq and the Kingdom of Saudi Arabia. As of 2018, Kuwait had a population of 4,621,638 people: 1,403,113 Kuwaitis and 3,218,525 expatriates [19]. The country has had an established Reference Mycology Laboratory since the 1970’s collecting good statistics on invasive candidiasis and aspergillosis [20,21], but figures for the annual incidence and prevalence of other fungal diseases is lacking. Here we attempted to estimate the burden of fungal infections at the national level for Kuwait, as has been done in several other countries in the Eastern Mediterranean region [22,23]. These estimates will be helpful for understanding the global impact of serious human fungal infections, and aid to raise awareness and promote the development of public health regarding aspects of fungal infections in the country. 

## 2. Materials and Methods 

### 2.1. Literature and Data Search

Laboratory diagnoses were collated from the Reference Mycology Laboratory in Kuwait. A search of government reports and scientific societies communications was performed, and the published literature on fungal infections in Kuwait was reviewed using the electronic search engines PubMed and Google Scholar in English. The following terms were used: “fungal infections + Kuwait”, “invasive fungal infections + Kuwait”, “aspergillosis/*Aspergillus* + Kuwait”, “severe asthma with fungal sensitization (SAFS) + Kuwait”, “candidemia/candidiasis/*Candida* invasive infections + Kuwait”, “Mucormycosis + Kuwait” and “tinea/dermatophytosis + Kuwait”. In addition, all pertinent literature was obtained and assimilated. 

### 2.2. Frequency Estimates

Incidence rates were estimated per 100,000 habitants, taking into account the Kuwait population census. Prevalence was calculated for the chronic forms of aspergillosis and the cases of tinea.

### 2.3. Data Estimation Sources

We estimated the burden of serious fungal infections based on the specific data available for Kuwait, as well as the extrapolation of data on individuals at risk in the country. The population and age distribution for 2018 was obtained from the Public Authority for Civil Information official statistics [19]. The rates of pulmonary disease (tuberculosis (TB) and asthma) were obtained from the WHO (2017 TB data) and Masjedi et al. [24,25]. HIV data were obtained from the Joint United Nations Program on HIV/AIDS (UNAIDS) [26]. Further statistics on bed capacity and admission rates were collected from the Statistical Division of the Ministry of Health. Chronic obstructive pulmonary disease (COPD) prevalence was taken from Masjedi et al., and annual hospitalisation percentages from Saudi Arabia (22.2%) [25,27]. The rate of invasive aspergillosis (IA) complicating COPD was taken from Spain (1.3%) [11]. Where no data existed, we estimated the burden of serious fungal infections based on underlying conditions using the model proposed by LIFE (Leading International Fungal Education) [28,29]. Chronic pulmonary aspergillosis (CPA), allergic bronchopulmonary aspergillosis (ABPA), and severe asthma with fungal sensitization were estimated in adults only. The cases of candidemia were calculated by estimating the number of cases in the intensive care unit (ICU) at a rate of 4 per 100,000 [30]. In the case of *Candida* peritonitis, we assumed that the rate was half of those ICU candidemia cases [31]. For the number oral candidiasis cases, we assumed that 90% of untreated HIV patients presenting with advanced HIV infection would have this type of candidiasis [32]. For oesophageal candidiasis, we assumed that 20% of patients not on antiretroviral therapy (ARVs), and 0.5% of those on ARVs, are affected annually [33]. To establish the prevalence of recurrent vulvovaginal candidiasis (VVC), an estimate of 6% of adult pre-menopausal women was used [34]. Prevalence and annual incidence rates were estimated per 100,000 inhabitants. The prevalence was calculated for the following: recurrent VVC, CPA, ABPA, and SAFS, and the remainder of the estimates were calculated as annual incidence.

### 2.4. Statistical Analysis

Descriptive statistics were used to summarize the data and expressed per 100,000 population. Estimated numbers over 100 were rounded to the nearest 10, and over 1000 to the nearest 1000. Sensitivity analysis was not done, as the base estimates are themselves based on extrapolated data, so it would not be unreliable. 

## 3. Results

Kuwait has an estimated population of 4,226,920 people: 1,303,246 are Kuwaitis and the remainder are expatriates (Table 1) [19]. Because of the expatriate work force, the population is disproportionally male of working-age; 63.6% are men, and 21.2% are children below the age of 15 years, with only 4.3% being elderly above 60 years. The female population was 1,230,068, and of these, only 7.1% are >60 years of age [19]. The total number of patients diagnosed with HIV was 414, and with pulmonary tuberculosis 986, and both rates were low. The prevalence of COPD in adult patients is estimated to be 226,289, of which 53,165 patients are admitted to hospital annually (Table 1). In addition, there were 158 cases of lung cancer in 2017. The estimation of serious fungal infections incidence and prevalence based on the population is shown in Table 2, with the number of infections per underlying disorder and the rate per 100,000 population.

### 3.1. Candidiasis

Candidemia and IA are the most frequent invasive infections. A recent report from the period 2006–2017 showed a total of 2075 isolates of six *Candida* species obtained from 1448 documented cases represented by a rate of mean annual incidence of 3.69/100,000 [20]. The annual incidence of candidaemia is 288 cases, a rate of 6.8 per 100,000 inhabitants. With regards to candidaemia cases, 123 (42.7%) cases had cancer or were immunocompromised, and 46 (16%) cases had had surgery or were in ICU. 

With regards to species distribution, *C*. *albicans* was the most frequent from 2006 to 2012, later replaced by *C*. *parapsilosis* in the next 4 years. This was probably explained by an ongoing outbreak in a neonatal ICU in the main maternity hospital in the country, which represented one-third of the collected isolates. The third and fourth most common species isolated were *C*. *tropicalis* and *C*. *glabrata*, respectively [20]. Resistance among *Candida* species appears to be still uncommon (<5%), but the trend is toward emerging fluconazole resistance, particularly in *C*. *albicans* and *C*. *parapsilosis*. In a previous study by Asadzadeh, a total of 442 *C. parapsilosis* isolates recovered from blood and other clinical specimens from nine hospitals in Kuwait during January 2012–December 2015 were examined, and 15 out of 442 (3.4%) *C. parapsilosis* isolates were resistant to fluconazole [4]. In a recent study, *C. auris* has become increasingly isolated and caused multiple cases with significant morbidity and mortality in Kuwait [35]. Moreover, a total of 158 *C. auris* isolates, over a 3.5-year period (May 2014 to September 2017), were obtained from 56 (31 males and 25 females) patients (age ranged from ≥13 to 89 years) [36]; of 56 *C. auris* isolates tested, all were resistant to fluconazole, 41/56 (73%) and 13/56 (23%) were additionally resistant to voriconazole and amphotericin B, respectively. 

With regards to *Candida* peritonitis and intra-abdominal candidiasis, an annual incidence of 150 cases representing a rate of 3.5 per 100,000 inhabitants is likely, not including cases of complicating chronic ambulatory peritoneal candidiasis. These comprise 123 (82%) cases with no underlying disease, 4 cases (2.7%) with cancer or were immunocompromised, and 23 (15.3%) cases having surgery or being in ICU.

Oral candidiasis is estimated in 71 patients with CD4 counts <200/µL, but we have not been able to estimate the incidence of oral candidiasis in non-HIV patients, likely to be much higher. Oesophageal candidiasis is estimated at 33, with a rate of 0.8/100,000, again not including non-HIV infected individuals. 

A prospective study conducted over 6 months at a tertiary care hospital in Kuwait to determine the prevalence of vulvovaginal candidiasis reported a total of 231 women who yielded *Candida* species with *C. albicans* predominance (73.9%) [37]. In Kuwait, the prevalence of recurrent vulvovaginal candidiasis (RVVC) in the adult female population is estimated at 54,842, nearly 4.5% of adult women without underlying disease—2595/100,000.

### 3.2. Aspergillosis

Invasive aspergillosis was estimated in leukaemia, lymphoma, myeloma, transplant recipients, lung cancer, and patients with COPD who are hospitalized. Many more renal transplants are done in Kuwait compared with other solid organ transplants. The number of patients that had acute myeloid leukaemia per year is 35, while there are 158 lung cancer cases each year. The COPD prevalence of 7.1% [25] and a hospitalization rate of 22.5% yield a risk population for invasive aspergillosis of 48,245 individuals, and a probable total number of IA cases of 704 (16.7 per 100,000), the vast majority related to COPD.

Although pulmonary tuberculosis is not common, COPD and asthma are relatively common, so we have estimated a prevalence of nearly 1000 individuals with chronic pulmonary aspergillosis, of whom 20% are related to TB. This could be an over-estimate, as the age demography is young in Kuwait and foreign workers with significant chest problems will be unlikely to work and stay in the country. 

Kuwait has a high asthma prevalence especially among Kuwaitis, with an average rate of 25.9% [25]. Khadadah et al. showed that the prevalence of asthma was 15% among Kuwaiti adults (93,923), the figure we have used, and 18% among children (70,158) [38]. The prevalence of asthma (actually shortness of breath assumed to be asthma) among immigrants is much lower, as described by Al-ayyadhi—around 7.6% [39]. We have therefore estimated the total asthma prevalence to be about 315,473 adults or 9.5% of the adult population overall.

The rate of ABPA in Kuwait is not known; however, Al-Mobeireek et al. from Saudi Arabia has estimated ABPA to be 2.7% [40]. Kuwait shares a similar demography, and with that, the estimated number of cases of is probably about 7889 with a rate of 187/100,000. The sensitization rate of *Aspergillus* among asthmatic individuals was 21.3% and was considered to be an important factor determining asthma severity in desert environments [41]. The estimated number of SAFS cases among inhabitants is Kuwait is 10,411 with a rate of 246/100,000, although this could be an over-estimate, as severe asthmatic foreign workers are unlikely to remain working in Kuwait for very long. 

### 3.3. Other Opportunistic Invasive Fungal Infections 

The current number of HIV/AIDS patients is 414, among which the number of diagnosed cases not receiving ARVs is 79. Therefore, the number of cases of opportunistic fungal infections in HIV patients in Kuwait will be in single figures annually and difficult to estimate. There will likely be many more cases of *Pneumocystis* pneumonia in HIV patients, but currently this is difficult to estimate. Mucormycosis, partly related to diabetes, which is common among Kuwaitis, is recorded and on a population basis, we estimate over 23 cases annually, 22 (95.7%) cases with no underlying diseases, and only 1 (4.3%) was immunocompromised or had cancer. 

### 3.4. Other Serious Fungal Infections 

We searched for information on infections caused by *Basidiobolus* and *Ramichloridrium* as well as fungal keratitis, sinusitis, tinea capitis, mycetoma, chromoblastomycosis, sporotrichosis, but found no data. However we have estimated the annual incidence of fungal keratitis based on data from Egypt (15.5/100,000) as a total of 654 cases [42]. 

## 4. Discussion

We adopted a systematic approach to estimate the number of common fungal infections in Kuwait, which is based on widely used methodology [43,44]. This was a challenging task due to the lack of some information regarding either fungal diseases and/or risk groups. 

Microscopy and culture have been used for fungi for over a century with few alterations in the techniques, other than gradual improvement in blood culture techniques. The first non-culture-based tests were introduced in the 1960’s (*Aspergillus* antibody) and 1970’s (cryptococcal and other antigen tests). Since then, the cryptococcal antigen has been perfected and re-configured as a point of care test, and *Aspergillus* and *Histoplasma* antigen tests are in routine use. Antibody testing for histoplasmosis, paracoccidioidomycosis, coccidioidomycosis, candidiasis, sporotrichosis, and blastomycosis have been gradually introduced but are not all supported by modern sensitive techniques or commercial platforms. Since the start of the new millennium, and particularly in the last 7 years, real-time polymerase chain reaction (PCR) tests to detect *Pneumocystis jirovecii*, *Aspergillus* spp., *Candida* spp., and *Trichophyton* spp. have been commercialized, providing more sensitivity and potentially faster results. The identification of positive culture can be done rapidly using matrix-assisted laser desorption/ionization time-of-flight mass spectrometry (MALDI-TOF MS) systems (especially for yeasts) or using sequencing. Kuwait has been the beneficiary of some of these tests, notably MALDI-TOF introduced in 2012 and T2Candida in 2019. 

The estimated candidemia burden varies from country to country, with the highest reported in Pakistan at 38,795 cases and the lowest in Trinidad and Tobago with cases as low as 87 [45,46]. Qatar, which is geographically and socioeconomically close to Kuwait, reported 288 cases [23]. A recent report from the country has benched Kuwait at a mean annual incidence of 3.69/10^5^ with 1448 cases in 12 years [17]. In this study, the incidence of candidemia was 6.8/100,000, in which *C. albicans* was the most common isolated species, as also found in other neighbouring countries such as Saudi Arabia, Qatar, United Arab Emirates, and Lebanon [21,44,47,48,49,50,51]. However, infections due to non-*albicans Candida* species have risen over the past decades, and a shift from *C. albicans* to other *Candida* species such as *C. glabrata*, *C. parapsilosis*, and *C. tropicalis* has occurred. This species shifting has led to gradual changes in the antifungal susceptibility profile such as azole antifungal agents and the rise of intrinsically resistant or multidrug-resistant species. The emergence of multidrug-resistant *C. auris* in Kuwait has been also detected in a single case in United Arab Emirates and in multiple cases in Oman and Saudi Arabia [36,47,52].

In this study, the rate of invasive aspergillosis was determined as 16.7/100,000, which is higher than that reported by surrounding countries. Qatar estimated the rate to be 0.6/100,000, while the rate in Saudi Arabia was 7.6/100,000 [23,53]. Epidemiological knowledge of invasive *Aspergillus infections* in Kuwait and the Middle East is limited due to negligible national surveillance data. Rapid diagnosis of IA using a more sensitive non-culture-based technique such as PCR is available for accurate identification but is limited in our region [54]. Our total estimation of IA was 705 cases annually. Of these cases, 4 (0.6%) were estimated in relation to respiratory disease, 9 (1.3%) cases in the context of cancer/immunocompromised patients, and 691 (98.2%) in the context of critical care/surgery. This estimate depends on the high proportion of patients admitted to hospital with COPD, 22.2% in Saudi Arabia, which is consistent with other country rates in 2010/11 (Egypt 20.3%, Jordan 20.3%, Lebanon 28.8%, Syria 21.9%, and UAE 40.9%) [9]. On the other hand, the rates of COPD in these populations probably underestimated the overall prevalence (varying from 1.9% to 5.4%), as hospitalisation rates could reflect only severe cases, and not be reflective of the whole COPD population, exaggerating invasive aspergillosis rates. An alternative approach was recently published and estimated 730 to 2189 IA cases, but very few of those diagnosed are established before death [9]. Chronic respiratory diseases, such as asthma and COPD, are prominent in Kuwait. In Kuwait, asthma has a high prevalence with an average rate of 25.9%, and the rate of SAFS per 100,000 inhabitants is 168.63 [11]. ABPA was accordingly assumed to occur at a rate of 187/100,000, higher than those in Egypt (162/100,000) and Iran (48.2/100,000) [55]. The total prevalence of CPA was estimated at 995 patients, only 20% amongst those who survived pulmonary TB in 2017. The high rates of asthma and COPD justify this estimation. 

Published rates of diagnosed vaginal yeast infections based on completed survey studies were limited. A total of 54,842 Kuwaiti women aged between 15 and 50 years were estimated to suffer from recurrent vulvovaginal candidiasis every year. Our rate was lower than the rate of vaginal candidiasis estimated in the Qatari population (3800/100,000) [23]. In a recent study from Saudi Arabia, in 205 out of 394 patients suffering from vaginal infections, *Candida* vaginitis episodes accounted for 58.5%, followed by 41% bacterial vaginitis, and 0.5% trichomoniasis infections [56].

The expatriate population in Kuwait represents two-thirds of the total population, and most of them are adult men (63.6%). The Gulf Health Council of the Gulf Cooperation Council (GCC) regional alliance proposed draft provisions to regulate the working program of the expatriate workforce in Gulf countries [57]. This program conducts all necessary medical tests and examinations for the expatriates within 2 months from the date of their arrival [57]. Regarding infectious diseases, all expatriates should have the following tests: HIV, hepatitis (B, C), tuberculosis, microfilaria, and malaria. Expatriates with any positive test, except for malaria, are unlikely to work and stay in the country.

Global warming could be one of the most significant factors that alters fungal populations. The warmer temperatures could change the distribution of heat-susceptible fungal species into thermotolerant species. This may raise the possibility of new fungal diseases [58,59]. In addition, global warming may increase the prevalence of thermophilic fungi that produce mycotoxins. This will pose another threat to human health [59]. *C. auris* could be the first pathogenic fungi emerging from climate change. It has been found to be thermotolerant if compared to other yeasts, besides its resistance to all major classes of antifungal drugs [60,61]. Another potential factor is increasing drug resistance in clinically relevant fungi due to excessive use in clinical, veterinary, and agriculture settings [59,62]. Azole fungicide use in the agricultural context has been identified as a potential cause for the development of resistance in *Aspergillus* spp. [63]. All these factors could increase fungal infections in the near future, and research is required on the global burden of fungal diseases and their effect on human health.

This estimation approach is necessarily a rough approximation to the actual annual incidence and prevalence of most diseases. One uncertainty is whether non-Kuwaitis were included in the large-population studies of COPD or not. Another relates to the lack of in-country data for many conditions. Only one reference laboratory is available to serve the entire country, with a limited number of clinical experts in fungal disease diagnosis and management. Additionally, fungal infections may be underdiagnosed due to the lack of optimum diagnostic tests. Specialized training for medical laboratory scientists is crucial to improve patient outcomes and inform public health.

## 5. Conclusions

This is the first study on the burden of serious fungal infections in Kuwait. According to our estimates, RVVC was the most common fungal infection, followed by SAFS. Our estimates also indicate that fungal infections are ignored in Kuwait, which requires more efforts of health care experts. Improving diagnostic tools and regular monitoring of the incidence of fungal infections are measures that could improve the accuracy of these estimates and lead to prompt identification and treatment.

## Figures and Tables

**Table 1:** Demographic and health data from Kuwait from groups of patients at risk for fungal infections.

Population Segment	Kuwaitis	Non-Kuwaitis	Total
Total population, 2018	1,303,246	2,923,674	4,226,920
Total adults, 2018	845,190	2,482,821	3,328,011
Adult women, 2018	431,208	798,860	1,230,068
Children (0–14)	458,056	440,853	898,909
Women over 60 years-old, 2018	48,087	39,431	87,518
HIV population			414
HIV population receiving ART *			355
Pulmonary tuberculosis, 2017			986
Asthma in adults, 2017	126,779	188,694	315,473
COPD ** in adults			236,289
COPD patients admitted to hospital			53,165
Lung cancer, 2017			158
Acute leukaemia, 2017 ***			15
Renal transplant recipients, 2014			80
Liver transplant recipients, 2014			4
Allogeneic stem cell transplant, recipients, 2017 ***			3
Heart transplant recipients, 2017	0	0	0
Lung transplant recipients, 2017	0	0	0
Critical care beds, 2018			223

* ART = Anti-retroviral treatment; ** COPD = Chronic obstructive pulmonary disease; *** Not including travel-abroad cases.

**Table 2:** Estimated burden of fungal infections.

Serious Fungal Infection	No Underlying Disease	HIV/AIDS	Respiratory Disease	Cancer + Immunocompromised	Critical Care + Surgery	Totals	Rate/100,000
Cryptococcal meningitis						0	0
*Pneumocystis* pneumonia		2		4		6	0.1
Invasive aspergillosis			4	9	691	704	16.7
Chronic pulmonary aspergillosis post TB (Prevalence)			199			199	4.3
Chronic pulmonary aspergillosis—all			995			995	21.3
Allergic bronchopulmonary aspergillosis (ABPA)			7887			7887	187
Severe asthma with fungal sensitisation (SAFS)			10,411			10,411	246
Candidaemia	119			123	46	288	6.8
*Candida* peritonitis	123			4	23	150	3.5
Oral candidiasis		71				71	1.7
Oesophageal candidiasis		33				33	0.8
Recurrent *Candida* vaginitis (>4×/year)	54,842					54,842	2595
Mucormycosis	22			1		23	0.5
Fungal keratitis	654					654	15.5
